# From Prediction to Intervention: Artificial Intelligence for Adaptive Response and Toxicity Modeling in Cellular Therapies for Hematologic Malignancies

**DOI:** 10.3390/cancers18162598

**Published:** 2026-08-12

**Authors:** Behzad Amoozgar, Ayrton Bangolo, Danielle C. Thor, Shibhani Rajanna, Shareif Abdelwahab, Ahmed S. Mohamed, Charlene Mansour, Syed Usman Ehsanullah

**Affiliations:** 1 Department of Bone Marrow Transplant and Cellular Therapy, Loma Linda University Health, Loma Linda, CA 92350, USA; 2 Department of Hematology and Oncology, Hackensack Meridian John Theurer Cancer Center, Hackensack, NJ 07047, USA; 3 Department of Blood and Marrow Transplant and Cellular Therapy, Fox Chase Cancer Center, Philadelphia, PA 19111, USA; 4 Department of Internal Medicine, Hackensack Meridian Health, Palisades Medical Center, North Bergen, NJ 07047, USA; 5 Department of Radiation Oncology, Cleveland Clinic Foundation, Cleveland, OH 44195, USA; 6 Rutgers New Jersey Medical School, Newark, NJ 07103, USA; 7 Department of Hematology Oncology, University Medical Center, El Paso, TX 79905, USA

**Keywords:** hematology, oncology, cellular therapy, CAR-T cell therapy, hematopoietic stem cell transplant, AI, machine learning

## Abstract

Hematologic malignancies, including acute myeloid leukemia, myelodysplastic syndromes, lymphoma, and multiple myeloma, are characterized by profound biological heterogeneity and highly dynamic treatment trajectories that conventional, static prognostic systems incompletely capture. Cellular therapies, such as chimeric antigen receptor T-cell therapy and hematopoietic stem cell transplantation, offer potentially curative options for relapsed or refractory disease, yet outcomes remain highly variable, and management decisions regarding conditioning intensity, lymphodepletion, immunosuppression, and toxicity surveillance continue to be largely protocol-driven rather than individually adapted. Artificial intelligence (AI) and machine learning (ML) have demonstrated substantial promise in diagnostic support, prognostic stratification, and multimodal data integration across hematologic malignancies, but existing models remain predominantly static and related to pre-treatment in orientation, limiting their utility for real-time clinical guidance. This review summarizes current AI applications in hematologic oncology; critically compares the strengths, limitations, and clinical applicability of major AI model classes, including traditional machine learning, deep learning, multimodal integrative frameworks, reinforcement learning, digital twins, and emerging foundation models and large language models; and proposes an adaptive, multimodal paradigm. We examine key enabling technologies and address the clinical, regulatory, ethical, and implementation challenges that must be resolved before these systems can be deployed at the bedside. We argue that the central challenge facing the field is no longer whether AI can predict outcomes, but whether it can actively guide real-time therapeutic decisions, and that achieving this transition will require interdisciplinary collaboration, prospective validation, and governance frameworks capable of ensuring interpretability, equity, and clinical trustworthiness.

## Simple Summary

Artificial intelligence is increasingly being used in hematologic malignancies to support diagnosis, risk prediction, and the integration of complex clinical and molecular data. However, most existing artificial intelligence models rely on information collected at a single point in time and remain largely investigational rather than integrated into routine clinical care. This limitation is particularly important for cellular therapies, including CAR T-cell therapy and hematopoietic stem cell transplantation, where disease status, treatment response, and the risk of complications can change rapidly throughout the treatment course. This review examines the current applications and limitations of artificial intelligence in hematologic malignancies and explores the transition toward adaptive systems that continuously integrate clinical, laboratory, genomic, imaging, and treatment data. Such approaches could ultimately support individualized decisions regarding treatment selection, conditioning and lymphodepletion, immunosuppression, toxicity prevention, and post-treatment monitoring. Prospective validation, interpretability, appropriate regulation, and careful integration into clinical workflows will be essential before these systems can reliably guide patient care.

## 1. Introduction

Hematologic malignancies, including acute myeloid leukemia (AML), myelodysplastic syndromes (MDS), lymphoma, and multiple myeloma, represent a diverse group of neoplasms characterized by considerable biological and clinical heterogeneity [1,2,3,4,5,6]. This heterogeneity, spanning distinct molecular pathways and an expanding array of targeted and novel therapeutic strategies, translates into wide variability in disease biology, treatment response, relapse risk, and survival outcomes across patients, even within the same diagnostic category [1,4,5,6]. Over the years, numerous international prognostic systems have been developed to address this complexity. The International Prognostic Index (IPI) and its updated revisions for lymphomas, and the International Prognostic Scoring System (IPSS), including its molecular revision (IPSS-M) for MDS, remain valuable tools for risk stratification [1]. However, studies have shown that patients within the same risk group can still experience markedly different clinical trajectories, indicating that these systems do not fully capture the dynamic and individualized nature of disease behavior [1,7]. This persistent gap in prognostic precision has created a need for more flexible, data integrative approaches capable of accounting for the complexity of hematologic malignancies.

The therapeutic landscape across these diseases is correspondingly heterogeneous and has expanded rapidly. In AML, intensive anthracycline-cytarabine induction is now complemented by hypomethylating agent plus venetoclax combinations for patients unfit for intensive therapy, and by mutation-directed agents including FLT3 inhibitors (midostaurin, gilteritinib, quizartinib) and IDH1/IDH2 inhibitors (ivosidenib, enasidenib, olutasidenib), with menin inhibitors emerging for KMT2A-rearranged and NPM1-mutated disease. In MDS, management spans erythropoiesis-stimulating agents and luspatercept for lower-risk disease through hypomethylating agents and allogeneic transplantation for higher-risk disease. In lymphoma, anti-CD20 monoclonal antibody-chemotherapy backbones have been supplemented by Bruton tyrosine kinase inhibitors, BCL2 inhibitors, antibody–drug conjugates such as polatuzumab vedotin and brentuximab vedotin, immune checkpoint inhibitors, and CD3 × CD20 bispecific antibodies, alongside investigational agents directed at the DNA damage response and PI3K signaling [4]. In multiple myeloma, proteasome inhibitors, immunomodulatory drugs, and anti-CD38 monoclonal antibodies form the therapeutic core, now extended by BCMA- and GPRC5D-directed bispecific antibodies and CAR T-cell products, while local modalities retain a role in selected frail patients with extramedullary disease [6]. Germline predisposition and familial aggregation add a further, inherited dimension to risk assessment that conventional prognostic indices do not capture [5]. Critically, this proliferation of biologically distinct, sequentially deployed therapies compounds rather than resolves the prognostic problem: as the number of available options and their possible sequences grows, so does the difficulty of determining which patient will benefit from which intervention, and at what point in the disease course.

The emergence of cellular therapies has substantially altered the treatment landscape for patients with relapsed or refractory hematologic malignancies. Chimeric antigen receptor (CAR) T-cell therapy represents a groundbreaking approach to cancer treatment, offering significant advancements in the management of hematologic malignancies [8]. Allogeneic hematopoietic stem cell transplantation (HSCT) remains the most important curative therapy for AML and can significantly improve survival [9]. However, the benefit of these interventions is variable; while some patients achieve durable responses, others relapse early or develop severe treatment-related complications. Relapse remains one of the most critical causes of transplant failure in patients with AML receiving HSCT [9]. CAR T-cell therapy carries risks of cytokine release syndrome (CRS) and immune effector cell-associated neurotoxicity syndrome (ICANS), while allogeneic HSCT is associated with graft-versus-host disease (GVHD), infections, and prolonged cytopenias. Across hematologic malignancies more broadly, bloodstream infection remains a significant cause of mortality, particularly amid rising antibiotic resistance [10]. Despite the high-stakes nature of these interventions, key management decisions, which include conditioning intensity, lymphodepletion strategies, immunosuppression, and toxicity prevention, remain largely standardized rather than individualized to the patient, even though patient risk evolves continuously over the course of treatment [11].

Artificial intelligence (AI) and machine learning (ML) have increasingly been applied to hematologic malignancies, with applications spanning outcome prediction, disease classification, biomarker discovery, and multi-omics data integration [1,2,7,12]. ML encompasses a wide range of approaches, including supervised and unsupervised learning, deep learning, and reinforcement learning, each with distinct strengths for pattern recognition in complex clinical and genomic datasets [2]. In a recent systematic review and meta-analysis by Alem et al., ML models demonstrated acceptable discriminative performance for predicting hematologic cancer outcomes, with pooled AUC values around 0.78–0.80, and externally validated top-performing algorithms outperformed standard regression models [1]. Multimodal AI frameworks are further expanding the ability to integrate clinical, genomic, imaging, pathology, treatment, and outcome data. MOSAIC, a multimodal AI framework designed for classification and personalized prognostic assessment in rare cancers, demonstrated that AI-based nonlinear approaches for survival prediction outperformed conventional statistical techniques and reference prognostic tools in MDS [13]. AI-assisted clinical frameworks integrating genomic and clinical data have also shown the capacity to improve point-of-care decision making and reveal clinically relevant translational insights [14]. These advances collectively support the potential role of AI in improving risk stratification beyond traditional prognostic systems.

Despite these promising results, several critical limitations constrain the current impact of AI in hematologic malignancies. Most existing models remain static, relying predominantly on baseline or pre-treatment variables, with limited incorporation of longitudinal data that capture the dynamic evolution of disease and treatment response over time [1,2]. Many studies exhibit a high risk of bias, and the discrimination ability between top-performing ML algorithms and standard regression models has not been consistently superior in internal testing sets [1]. Reporting of model calibration and clinical utility remains inadequate across much of the literature [1]. Furthermore, despite several reports and the achievement of promising results, none of the ML-based pipelines have been directly translated into clinical practice [2]. A large percentage of hematologists have limited familiarity with these tools, which can cause skepticism, and concerns have been raised by patients regarding privacy, reliability of outcomes, and loss of human interaction [2]. Recommendations for standardized implementation, such as those proposed for AI in clinical flow cytometry, including guidance on validation, revalidation, computational considerations, and regulatory frameworks, highlight the persistent gap between model development and real-world clinical deployment [15]. Importantly, several reviews note limited clinical translation and workflow integration, meaning that AI in hematologic malignancies has largely remained a tool for prediction rather than for real-time clinical intervention.

These limitations provide the rationale for the framework proposed in this review; adaptive, multimodal AI systems that integrate longitudinal clinical, laboratory, genomic, treatment, and toxicity data to update risk assessment over time. In the setting of cellular therapy, such models may eventually help personalize conditioning intensity, lymphodepletion strategies, immunosuppression, toxicity prevention, monitoring intensity, and early intervention.

To realize this, emerging multimodal frameworks have demonstrated the feasibility of integrating diverse data types, while synthetic data generation using generative AI has shown the capacity to address data sparsity, resolve incomplete information, augment limited datasets, and preserve patient privacy, thereby accelerating research and precision medicine in hematology [13,16]. Federated learning approaches further enable broad clinical application of AI models while guaranteeing data protection [13]. This review summarizes current AI applications in hematologic malignancies, critically appraises the limitations of existing predictive models, and proposes a framework for adaptive, multimodal, temporally dynamic AI systems designed to support response and toxicity modeling in cellular therapies for hematologic malignancies.

### 1.1. Review Methodology and Scope

This is a narrative, expert-informed review rather than a systematic review; no formal PRISMA protocol or prospective registration was used, consistent with its exploratory and conceptual aims. Searches were conducted iteratively between January 2025 and May 2026 and covered literature indexed through May 2026, without a lower date restriction; English-language publications were included, and non-English-language publications without an English translation were excluded. Relevant literature was identified through structured searches of PubMed/MEDLINE, Embase, and Google Scholar using combinations of terms including “artificial intelligence,” “machine learning,” “deep learning,” “foundation models,” “large language models,” “hematologic malignancies,” “leukemia,” “lymphoma,” “myelodysplastic syndrome,” “multiple myeloma,” “chimeric antigen receptor T-cell therapy,” “hematopoietic stem cell transplantation,” and “clinical decision support,” supplemented by hand-searching the reference lists of relevant reviews and primary studies. Priority was given to peer-reviewed systematic reviews, meta-analyses, and studies reporting externally validated model performance; preprints and conference abstracts were included selectively where they represented the most current available evidence on a rapidly evolving subtopic (e.g., foundation models and large language models in hematology). We did not apply formal risk-of-bias or quality-appraisal tools to individual primary studies; where relevant, we instead draw on published systematic reviews and meta-analyses that have already conducted such formal appraisal (e.g., Alem et al. [1]). This review’s central contribution is the synthesis of the static-versus-dynamic modeling literature into a proposed adaptive, multimodal AI framework specific to cellular therapy, and it should accordingly be read as a conceptual and critical synthesis rather than an exhaustive systematic review.

### 1.2. Positioning Relative to Existing Reviews

Several recent reviews have summarized AI applications in hematologic malignancies from complementary vantage points. Shouval et al. provided an early foundational overview of machine learning methodology in hematology [17]; El Alaoui et al. subsequently mapped current practices and future prospects for AI across hematology more broadly [7]; and more recent syntheses, including Almaghrabi’s narrative review spanning diagnostic precision to therapeutic innovation [18], Kilic Gunes’s survey of current trends and application areas [19], and Nazha et al.’s 2025 *Blood * review [20], have each expanded the catalogue of reported applications as the field has matured. Most recently, Li et al. explicitly framed the field’s trajectory “from static assessment to dynamic precision management” [21], a framing that closely parallels the central thesis of the present review.

**Figure 1:**
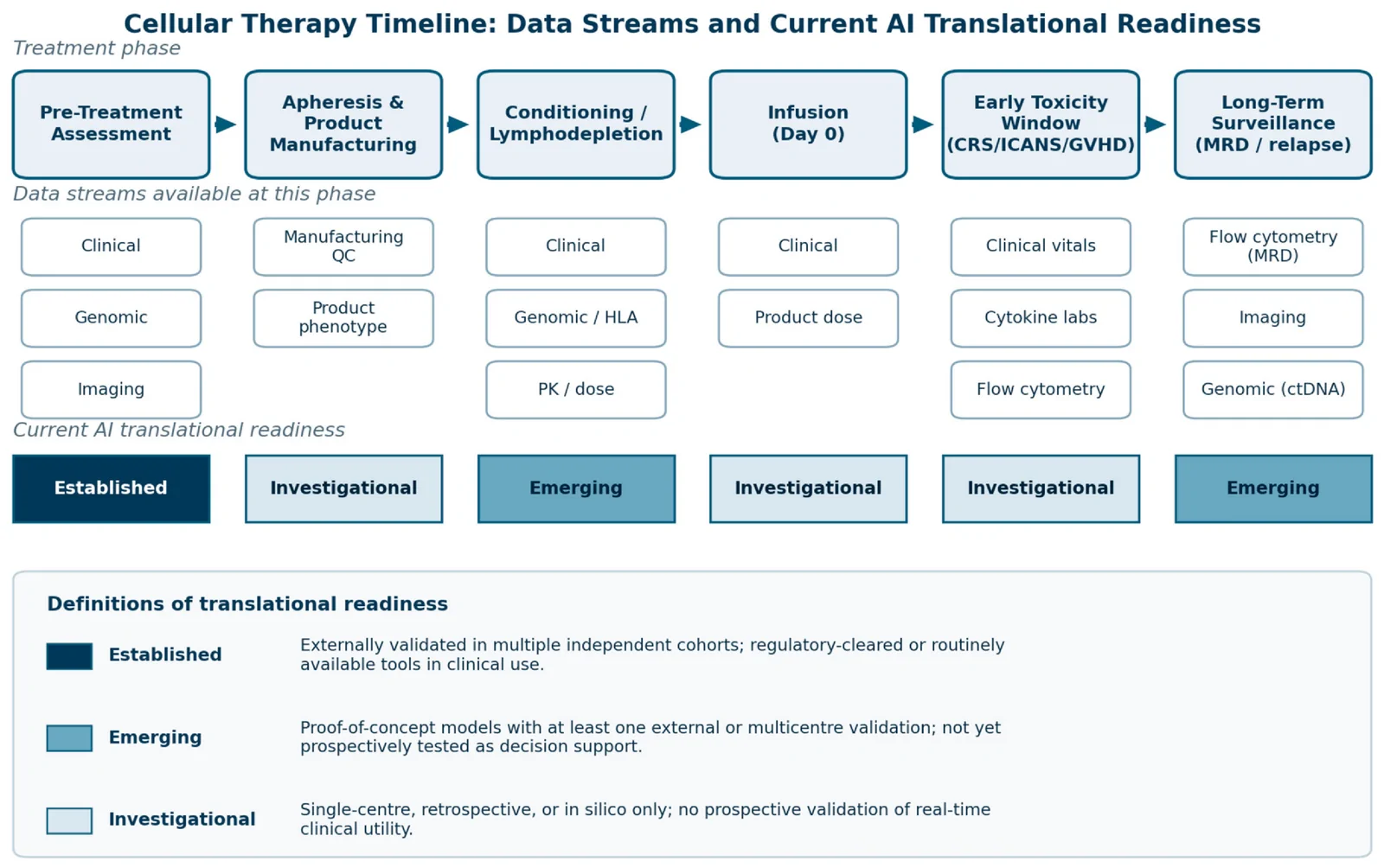
Conceptual framework mapping the cellular therapy treatment timeline—from pre-treatment assessment and product manufacturing through conditioning/lymphodepletion, infusion, the early toxicity window, and long-term surveillance—against the clinical, genomic, imaging, pharmacokinetic, flow cytometric/MRD, and manufacturing data streams available at each phase. The lower band grades the current translational readiness of AI models at each stage as established, emerging, or investigational according to the definitions shown; grading was derived from the model-class appraisal in Table 1 and the benchmarking data in Table 2. The figure is intended to make explicit the mismatch between the phases at which data are richest and continuously generated (the peri-infusion and early toxicity windows) and the phases at which validated AI decision support currently exists (predominantly pre-treatment).

What distinguishes the current work from these antecedents is not merely an updated inventory of published models, but (i) an explicit, side-by-side comparative appraisal of the relative strengths, limitations, and translational readiness of distinct AI model classes (Table 1), rather than a disease- or technique-siloed inventory; (ii) a specific focus on cellular therapy, in which the sequential, time-dependent nature of conditioning, lymphodepletion, immunosuppression, and toxicity management is used as the organizing framework for evaluating AI readiness, rather than treating cellular therapy as one application area among many; and (iii) explicit incorporation of the most recent generation of foundation models and large language models, an area only briefly mentioned, if at all, in reviews predating 2025. In this sense, the present review is intended to complement rather than duplicate existing summaries, using cellular therapy as a stress test for the field’s overall readiness to move from prediction to intervention. The proposed conceptual framework and its relationship to the cellular therapy treatment timeline are summarized in Figure 1.

**Table 1:** Comparative Critical Appraisal of Major AI/ML Model Classes in Hematologic Malignancies and Cellular Therapy.

Model Class	Representative Examples	Strengths	Key Limitations	Clinical Applicability (Current Stage)	Principal Remaining Challenges
Traditional ML/regression-augmenting models	Alem et al. meta-analysis [1]; Nazha et al. random survival forest [22]	Interpretable; modest data requirements; pooled AUC 0.78–0.80, top performers up to 0.87 [1]	Static/baseline-only; superiority over regression inconsistent on internal testing [1]	Closest to translation; retrospective risk stratification	Prospective validation; calibration reporting; external replication
Deep learning/CNN imaging & digital pathology	CNN classification of smears/marrow/WSI [12]; lymphoma subtyping [3]	High fidelity in image pattern recognition; reduces inter-observer variability [3]	Black-box; large annotated datasets required; staining/scanner variability [15]	Emerging diagnostic support	Explainability; external validation; regulatory clearance
Multimodal integrative frameworks	MOSAIC [13]; oncology multimodal frameworks [23,24]	Integrates clinical + genomic + imaging; outperforms unimodal tools; federated + XAI-compatible [13]	Data completeness/missingness; complex validation; computationally intensive	Proof-of-concept, primarily MDS	Harmonized data pipelines; generalizability
Reinforcement learning/sequential decision models	Q-learning for GVHD prophylaxis [25,26]	Suited to sequential, adaptive decisions (immunosuppression, conditioning)	Confounding by indication; limited prospective testing	Exploratory/registry-based; no prospective trial in cellular therapy	Trial design; safety guardrails; off-policy evaluation bias
Digital twins	Preclinical oncology models [27,28,29]	Simulates individualized trajectories, multi-omic integration	Almost entirely preclinical; heavy infrastructure requirement	Investigational only	Validation methodology; scalability; regulatory pathway
Foundation models & large language models	Foundation models in clinical oncology [30]; LLM-based CDSS, CliCARE [19,31]	Pretrained on large-scale data; adaptable across downstream tasks; can synthesize unstructured longitudinal EHR data [30,31]	Inconsistent performance in oncology-facing CDSS [31]; minimal hematology-specific validation; black-box reasoning	Earliest stage; trending application, largely untested prospectively [19]	Need for prospective hematology validation; explainability; governance; regulatory oversight

**Table 2:** Quantitative Benchmarking of Reported Model Performance Across Representative Studies.

Study	Task	Reported Quantitative Benchmark	Comparator	Ref.
Alem et al. (meta-analysis)	Outcome prediction, pooled across studies	Pooled AUC 0.78–0.80; externally validated top performers AUC 0.87 (treatment-related complications)	Standard regression models	[1]
Bobak et al.	Dynamic mortality risk, MDS	AUC ~ 0.80 (independent validation)	Static baseline models	[32]
MOSAIC (D’Amico et al.)	Multimodal prognostic classification, MDS	Superiority reported qualitatively; no pooled AUC stated	Conventional statistical/reference tools	[13]
PKU-AML (Fan et al.)	Post-transplant relapse prediction	Accuracy reported as exceeding clinical judgment; no numeric AUC stated	Unaided clinician prediction	[9]
Shimomura et al.	Conditioning intensity (RIC vs. MAC)	Significant PFS difference reported; no discrimination statistic stated	Categorical MAC/RIC assignment	[33]
Nazha et al.	Personalized survival/risk, MDS	Dynamic probability updating demonstrated; no pooled AUC stated	No formal comparator stated	[22]

## 2. Current Application of Artificial Intelligence in Hematologic Malignancies

### 2.1. The Expanding Role of AI in Hematologic Oncology

As outlined in Section 1, hematological malignancies exhibit considerable heterogeneity in genomic architecture, treatment response, and survival outcomes, and established prognostic indices such as the IPI and IPSS/IPSS-M capture that heterogeneity incompletely [1]. Artificial intelligence has accordingly been proposed as a means of integrating clinical, molecular, and genomic data into individualized therapeutic strategies. This section surveys where AI has actually been deployed across the diagnostic and prognostic workflow; Section 3 then examines why those applications have not yet translated into real-time clinical decision support.

Machine learning (ML) is a type of AI that combines a broad range of computational techniques to identify complex patterns within datasets [12]. ‘Deep learning’ refers to a network of neural layers within ML [12], whilst convolutional neural networks (CNNs) refer to a type of ML that optimizes deep learning for analyzing images. These tools are increasingly applied to diagnose malignancies; analyze images, digital pathology specimens, and biomarkers; and monitor measurable residual disease (MRD) [7].

### 2.2. Imaging and Digital Pathology

The role of AI has historically focused on interpreting digital images and pathology. In this connection, CNNs have demonstrated high fidelity in interpreting peripheral blood smears, bone marrow aspirates, and histopathologic whole-slide images [12]. AI has also demonstrated particular utility in diagnosing lymphoma. Critically, it overcomes the inherent disparity amongst practitioners that arises when diagnosing lymphoma on the basis of complex histopathologic, immunophenotypic, and molecular data. Indeed, some have noted that lymphoma diagnosis may demonstrate substantial variability outside specialized referral centers, highlighting the potential value of AI-assisted diagnostic support systems to reduce this variability [3]. Moreover, deep learning models applied to whole-slide imaging have demonstrated the ability to classify lymphoma subtypes, identify high-risk molecular phenotypes, and extract morphologic features associated with prognosis and therapeutic response.

Imaging-based ML has also expanded rapidly across hematologic malignancies. ML systems applied to PET/CT and MRI imaging can extract quantitative radiomic features associated with disease burden, treatment response, and survival outcomes. In multiple myeloma specifically, AI-assisted imaging analysis has proven useful in detecting lesions, assessing marrow infiltration, and risk stratification [7]. With that said, AI’s future trajectory in hematologic malignancies transcends its traditional circumscribed role in diagnosing cancer. Rather, it extends to guiding clinical decisions by evaluating disease progression and response to treatment. This is particularly relevant in prognostication and high-risk therapeutic settings such as cellular therapy and stem cell transplantation, where patient risk evolves continuously over time.

### 2.3. Prognostication, Genomics, and Multimodal Modeling

The rapid expansion of genomic profiling in hematology has generated highly complex multidimensional datasets that increasingly require computational integration. AI-based approaches have therefore become particularly important for genomic classification, biomarker discovery, and prognostic modeling.

ML systems are capable of integrating complex variables such as transcriptomics and cytogenetics into multidimensional prognostic frameworks that extend beyond traditional linear statistical models. One important example is the MOSAIC framework, which is a multimodal AI platform developed to provide individualized classification and prognostic assessment in rare cancers and MDS. MOSAIC integrates clinical and genomic variables using nonlinear survival modeling approaches to provide superior prognostications compared with conventional statistical techniques and established prognostic systems [13].

The growing role of AI in prognostic modeling is further supported by the systematic review and meta-analysis conducted by Alem et al., in which ML models demonstrated acceptable discriminatory performance for predicting hematologic malignancy outcomes, with pooled AUC values ranging approximately from 0.78 to 0.80. Externally validated top-performing ML algorithms demonstrated improved predictive performance compared with conventional regression-based models, supporting the potential role of AI in refining prognostic precision [1].

Beyond this, multimodal AI frameworks are increasingly capable of integrating genomic, imaging, pathology, laboratory, and clinical outcome data into unified predictive systems. These approaches may ultimately support more individualized therapeutic strategies and dynamic longitudinal risk assessment.

### 2.4. Flow Cytometry and MRD Monitoring

Flow cytometry remains central to the diagnosis, classification, and monitoring of hematologic malignancies, particularly leukemia and lymphoma. However, manual gating and interpretation remain labor-intensive and highly dependent on expert review. AI-assisted flow cytometry systems have therefore emerged as promising tools for automated multidimensional analysis [15].

These systems may improve reproducibility, accelerate case prioritization, reduce human error, and identify subtle abnormal cellular populations that may otherwise be overlooked. AI-assisted MRD assessment is particularly important because longitudinal disease monitoring increasingly influences therapeutic decision-making, relapse prediction, and post-treatment surveillance [15]. Beyond diagnosis and disease classification, AI is increasingly being explored in therapeutic risk stratification and treatment personalization, particularly in the setting of high-risk cellular therapies.

### 2.5. Cellular Therapy and Transplantation

As introduced in Section 1, cellular therapies have substantially altered the treatment landscape for relapsed or refractory hematologic malignancies, yet clinical outcomes remain highly variable: relapse is a principal cause of treatment failure following allogeneic HSCT in AML, and CAR T-cell therapy carries substantial risks of cytokine release syndrome (CRS) and immune effector cell-associated neurotoxicity syndrome (ICANS), compounded by graft-versus-host disease (GVHD), infection, and prolonged cytopenias [8,9]. What is relevant to the present discussion is that this variability arises in a setting in which clinical, laboratory, and molecular data are generated continuously across the treatment course, making cellular therapy an unusually favorable, and unusually demanding, target for AI-based risk modeling.

Despite the high-risk nature of these interventions, many treatment decisions—including conditioning intensity, lymphodepletion strategies, immunosuppression protocols, toxicity prevention, and monitoring intensity—remain relatively standardized rather than dynamically individualized. This has generated increasing interest in adaptive AI systems capable of integrating longitudinal clinical, laboratory, genomic, treatment, and toxicity data to support real-time risk assessment and individualized therapeutic decision-making throughout the treatment course.

## 3. Limitations of Current AI Models in Hematologic Malignancies

In this Section, we briefly highlight the limitations affecting the use of AI in hematological malignancies. These limitations support the need for next-generation AI frameworks capable of integrating longitudinal clinical, laboratory, genomic, treatment, and toxicity data into adaptive multimodal systems. At present, most AI systems remain optimized for narrow prediction tasks rather than continuous integration into real-time therapeutic workflows. Consequently, there exists a significant gap between AI’s potential performance and actual clinical application.

Rather than functioning solely as static diagnostic and prognostic tools, future AI systems may eventually support dynamic risk assessment, individualized toxicity prevention, adaptive immunosuppression strategies, and real-time therapeutic intervention throughout the course of treatment in hematologic malignancies.

### 3.1. Static Nature of Current Predictive Models

Despite substantial advances in AI-based prognostic modeling, most currently available systems remain fundamentally static. Existing models rely predominantly on pretreatment variables and incorporate limited longitudinal information reflecting the evolving nature of disease biology, treatment response, toxicity, and relapse risk over time. This limitation is particularly important in cellular therapies, wherein risk changes dynamically throughout treatment. Current AI systems largely function as predictive tools rather than adaptive clinical decision-support frameworks capable of continuously updating individualized risk assessments during therapy. Consequently, the role of AI in real-time therapeutic intervention remains underdeveloped [1]. This static orientation is compounded by a second, related problem: even where models incorporate rich baseline data, their performance frequently fails to generalize beyond the population and institution in which they were developed.

### 3.2. Limited Generalizability

A major challenge in hematologic AI research involves the heterogeneity of clinical datasets and the limited generalizability of published models. Many studies remain retrospective, single-center investigations involving relatively small patient cohorts. Substantial variability exists across institutions in pathology workflows, blood smear preparation, bone marrow staining, imaging platforms, flow cytometry panels, sequencing technologies, toxicity grading systems, and supportive care practices. These differences may significantly affect AI model performance when applied outside the original development environment [15]. Consequently, strong internal validation frequently fails to translate into real-world clinical performance.

This limitation is particularly problematic in studies of cellular therapy, in which institutional differences in transplant protocols, immunosuppression strategies, and toxicity management may substantially affect outcome modeling and risk prediction.

Another major obstacle affecting the development of AI in hematological malignancies is limited access to sufficiently large and representative datasets, particularly in rare malignancies such as MDS [16]. This limitation is further compounded by important methodological concerns. Many studies, for example, demonstrate bias, whilst others lack a robust assessment of the clinical utility of using AI for a specific purpose [1]. These in turn limit the generalizability of such studies and the utility of using the study results to train AI accordingly. For these reasons, relatively few AI technologies have been directly integrated into routine clinical practice. These sample-size and generalizability constraints are further entangled with a distinct, though related, set of methodological weaknesses in how existing studies are designed, validated, and reported.

### 3.3. Methodological Limitations and Risk of Bias

The current hematologic AI literature remains limited by important methodological concerns. Alem et al. demonstrated that many studies exhibited high risk of bias, incomplete reporting of calibration metrics, and inadequate assessment of clinical utility [1]. Although ML models generally achieved acceptable discriminatory performance, superiority over traditional regression-based approaches was not consistently demonstrated across internal testing datasets.

Overfitting additionally remains a significant concern, particularly in studies involving relatively small datasets with large numbers of highly correlated variables. As a result, models may perform well within training cohorts while demonstrating reduced reproducibility in external populations. Collectively, these limitations help explain why AI in hematologic oncology has largely remained investigational despite strong retrospective predictive performance. A further, closely related barrier to clinical adoption concerns not how models are validated, but how their outputs are communicated and understood by clinicians.

### 3.4. Lack of Interpretability and Integration

Many advanced deep learning systems generate predictions without clearly explaining the reasoning underlying those outputs [3]. These ‘black-box’ models lack transparency, which may reduce clinician trust and complicate implementation in hematologic oncology, where treatment decisions frequently carry substantial toxicity and survival implications. Efforts to improve interpretability through explainable AI methodologies, including SHAP analysis and feature attribution frameworks, represent important advances [13]. Nevertheless, balancing predictive complexity with clinical interpretability remains an ongoing challenge.

Importantly, despite numerous reports demonstrating promising predictive performance, few AI-based systems have been directly integrated into routine clinical practice. Recommendations proposed for AI implementation in clinical flow cytometry emphasize the importance of continuous validation, revalidation, quality assurance, computational infrastructure, and regulatory oversight [15]. These recommendations highlight the persistent gap between experimental model development and practical real-world deployment. Beyond these technical and methodological barriers, however, clinical adoption ultimately depends on human, ethical, and regulatory factors that operate independently of model performance itself.

### 3.5. Human Factors, Ethical Concerns, and Regulatory Challenges

Human factors remain important barriers to implementation. Few hematologists and oncologists have sufficient familiarity with ML systems, which may entrench preconceived notions and skepticism regarding their reliability and usefulness [2]. The interpretability problem described in Section 3.4 compounds this: clinicians asked to act on a recommendation they cannot interrogate are unlikely to do so in a setting where treatment decisions carry substantial toxicity and survival implications [3].

Patients have additionally raised concerns regarding privacy, reliability of AI-generated recommendations, and the potential loss of human interaction in clinical care. Ethical and legal concerns surrounding data ownership, informed consent, algorithmic bias, accountability, and regulatory oversight remain incompletely resolved, particularly as AI systems become increasingly adaptive and capable of continuous learning from evolving clinical datasets.

### 3.6. Comparative Critical Appraisal of Current AI Model Classes

Although Section 3.1, Section 3.2, Section 3.3, Section 3.4 and Section 3.5 outline limitations affecting AI in hematologic malignancies broadly, these limitations are not distributed evenly across model types, and a more granular, comparative appraisal is needed to contextualize each model class’s readiness for clinical translation. Table 1 summarizes the principal strengths, limitations, and clinical applicability of the major AI model classes currently applied in hematologic malignancies and cellular therapy.

Traditional machine learning approaches, including regression-augmenting algorithms, random forests, gradient boosting, and random survival forests, remain the most extensively validated and interpretable model class. In the meta-analysis by Alem et al., externally validated top-performing ML algorithms achieved a pooled AUC of 0.87 for treatment-related complications, outperforming standard regression; however, this superiority was not consistently observed within internal testing cohorts, underscoring that apparent discriminative advantage may not persist without external validation [1]. These models’ relative interpretability and modest data requirements place them closest to near-term clinical translation, but their largely static, baseline-only design limits applicability to the longitudinal decision-making inherent to cellular therapy.

Deep learning and convolutional neural network-based models have demonstrated particular strength in image-intensive tasks, including peripheral blood smear interpretation, bone marrow morphology, and lymphoma subtyping, where they can reduce diagnostic variability outside specialized referral centers [3,12]. However, these models require large, well-annotated datasets, remain vulnerable to institutional variability in staining and imaging platforms, and function largely as black-box systems, limiting both generalizability and clinician trust [15].

Multimodal integrative frameworks, exemplified by MOSAIC, represent a meaningful advance by combining clinical, genomic, and outcome data within nonlinear survival models, outperforming conventional statistical and reference prognostic tools while incorporating federated learning and explainability methods [13]. Beyond hematology, multimodal integration of radiology, histology, genomic, and clinical data has been shown to improve predictive performance and enable discovery of novel biomarkers, though dataset size and completeness remain limiting [23,24]. Within hematology specifically, such frameworks remain confined largely to proof-of-concept validation in a small number of diseases (chiefly MDS to date) and require harmonized, multimodal data pipelines that few institutions currently possess.

Reinforcement learning approaches, particularly Q-learning applied to GVHD prophylaxis and immunosuppression tapering, offer a methodologically distinct advantage: the capacity to model sequential, time-dependent treatment decisions rather than single-time-point predictions [25,26]. This makes RL conceptually well matched to cellular therapy, where decisions must adapt continuously. However, RL models trained on registry data are vulnerable to confounding by indication, and no RL-guided intervention has yet been prospectively tested in a clinical trial of cellular therapy.

Digital twin technologies remain the least clinically mature of the model classes reviewed, with applications in hematologic malignancies confined almost entirely to preclinical or conceptual demonstrations [27,28,29]. Their considerable data and computational infrastructure requirements, combined with an absence of validated clinical use cases, place them furthest from bedside application among the approaches considered here.

Finally, foundation models and large language models (LLMs) constitute the newest and least hematology-specific model class; their architecture and hematology-specific limitations are examined in detail in Section 4.3 and are not repeated here. For the purposes of this appraisal, it suffices to note that they offer the broadest cross-task generalizability of any class considered, while having the least hematology-specific validation, and that no prospective evaluation within a cellular therapy workflow has yet been reported [19,30,31].

Considered together, these model classes are not competing alternatives but occupy complementary niches along a translational continuum: traditional ML and CNN-based imaging models are furthest advanced toward integration into narrow diagnostic and prognostic tasks, multimodal and reinforcement learning frameworks are approaching proof-of-concept validation for integrated and sequential decision-making, and digital twins and foundation models/LLMs remain predominantly investigational. This comparative framing clarifies that no single model class is currently positioned to deliver real-time, adaptive clinical decision support in cellular therapy; rather, next-generation systems will likely need to combine the interpretability and validated performance of traditional ML, the multimodal integration capacity of frameworks such as MOSAIC, the sequential decision-making logic of reinforcement learning, and the generalizable representation learning offered by foundation models.

Beyond the qualitative appraisal in Table 1, direct quantitative benchmarking across studies remains constrained by inconsistent outcome reporting. Table 2 summarizes the standardized discrimination statistics (area under the receiver operating characteristic curve, AUC) explicitly reported for the representative studies discussed throughout this review, alongside instances in which only qualitative superiority was reported.

**Figure 2:**
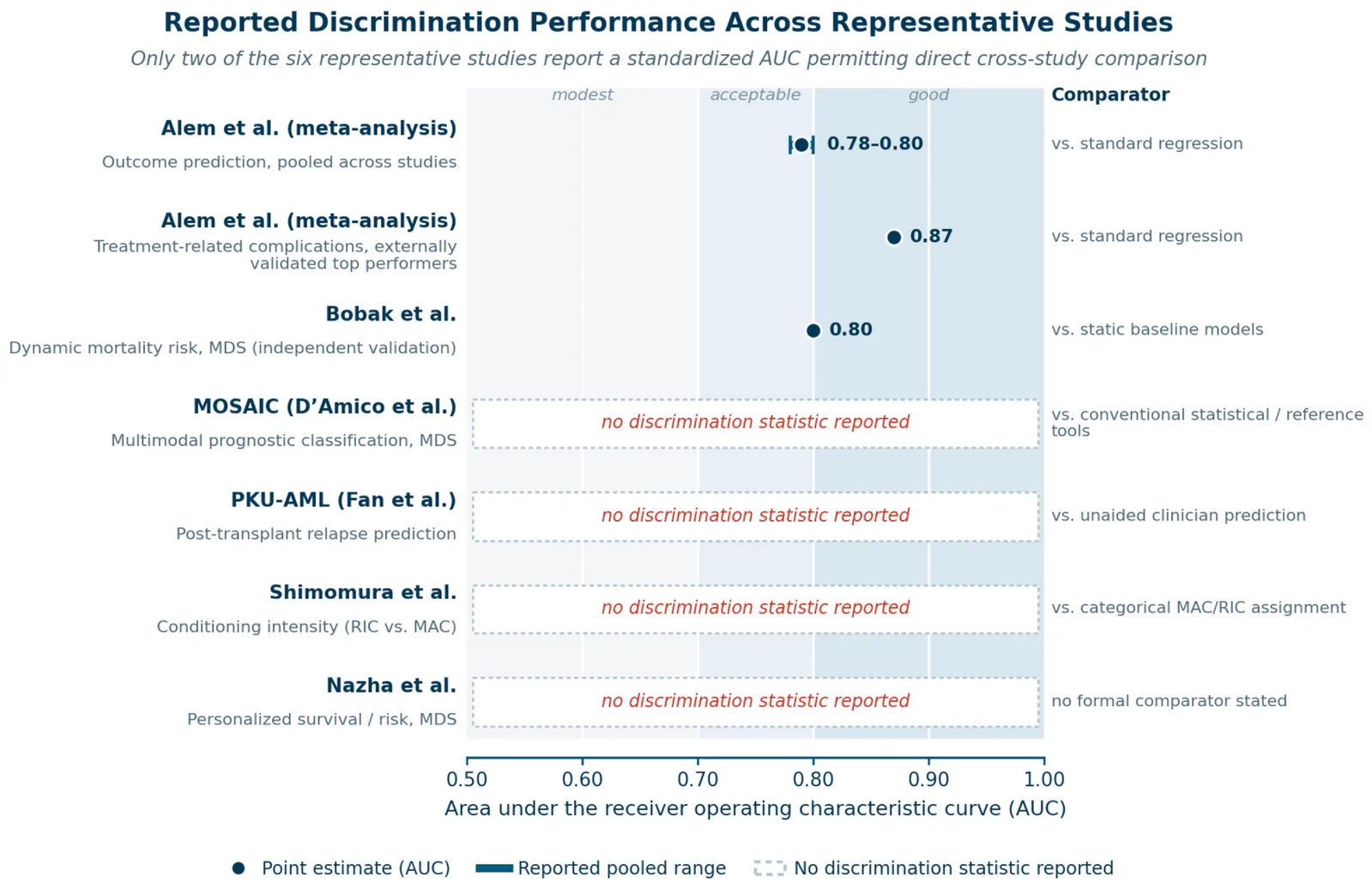
Benchmarking of reported discrimination performance across the representative studies summarized in Table 2 [1,9,13,22,32,33]. Filled markers denote reported point estimates of the area under the receiver operating characteristic curve (AUC); the horizontal bar denotes the pooled range reported in the meta-analysis by Alem et al. Dashed rows indicate studies for which no standardized discrimination statistic was reported, precluding direct cross-study comparison. Comparators differ substantially across studies and are shown at right. The figure is therefore intended to display the heterogeneity and incompleteness of current performance reporting in this literature, and not to rank models against one another; formal head-to-head benchmarking is not currently possible from the published data.

Only two of the six representative studies discussed in this review report a standardized discrimination statistic (AUC) permitting direct cross-study comparison (Figure 2); the remainder report qualitative or study-specific superiority claims, underscoring the calibration- and comparability-reporting gap already noted in Section 3.3 and reinforcing the need for standardized benchmarking frameworks (e.g., TRIPOD + AI [34]) going forward.

## 4. Emerging Paradigm: Dynamic and Multi-Modal Artificial Intelligence

### 4.1. Transition from Static to Dynamic AI Models

Conventional prognostic models in hematologic malignancies rely predominantly on variables captured at a single time point (typically at diagnosis) and therefore fail to capture the dynamic evolution of disease biology, treatment response, and toxicity over time [2,35]. This is a critical limitation in diseases such as myelodysplastic syndromes (MDS) and acute myeloid leukemia (AML), where disease trajectories are highly heterogeneous and evolve in response to therapy [2,36]. Machine learning (ML) offers the potential to refine risk stratification and improve prediction of clinical outcomes, but the majority of published ML-based pipelines have not been directly translated into clinical practice, in part because they remain static in design [2,17].

The need for incorporation of longitudinal data streams is increasingly recognized. Serial laboratory measurements, treatment response trajectories, and evolving molecular and immune profiles all contain clinically important temporal signals that static models cannot capture [2,17,35]. Time-dependent modeling approaches are increasingly explored in clinical machine learning, with the potential to continuously update risk estimates as new patient data become available [17,35]. The growing volume and complexity of digitized health data, including electronic health records, genomic data, and imaging, further necessitate ML approaches capable of processing high-dimensional, longitudinal datasets beyond the scope of traditional statistical methods [35]. At the same time, limited familiarity with ML concepts among clinicians remains an important barrier to adoption in hematology [17].

Concrete examples of dynamic modeling in hematologic malignancies are now emerging. Bobak et al. developed a dynamic mortality risk prediction model for MDS using longitudinal clinical data, achieving AUC values of approximately 0.80 in independent validation cohorts and outperforming static baseline models [32]. Similarly, Nazha et al. constructed a personalized prediction model using random survival forests trained on clinical and molecular data, demonstrating the ability to dynamically update survival and leukemia transformation probabilities at successive clinical time points [22].

Recurrent neural networks (RNNs), particularly long short-term memory (LSTM) architectures, are well suited for processing sequential clinical data with irregular time intervals, as commonly encountered in electronic health records [17,35]. Hoffmann et al. further demonstrated that optimized longitudinal data collection strategies can significantly improve predictive accuracy, underscoring the importance of data quality alongside model development [37].

### 4.2. Multi-Modal Data Integration

Hematologic malignancies are characterized by complex, multi-layered biology that cannot be fully captured by any single data modality. Multi-modal AI frameworks that integrate heterogeneous data types, including clinical variables, genomic and transcriptomic features, imaging and radiomic data, flow cytometry, and laboratory signals, enable improved prediction and classification by capturing cross-scale biological interactions [17,36].

The integration of multi-modal data is essential for precision oncology approaches in heterogeneous malignancies. Bode and Dong emphasized that precision oncology relies on the identification of key molecular alterations and their associated signaling pathways, and that advanced analytical approaches capable of integrating multi-dimensional data are critical for overcoming tumor heterogeneity [36]. Similarly, Shouval et al. highlighted that the integration of diverse data sources, such as imaging, pathology, omics, and clinical parameters, is fundamental for building robust machine learning models in hematology [17].

In oncology more broadly, multi-modal data integration has emerged as a central strategy for precision medicine. Lipkova et al. described how artificial intelligence methods can integrate radiology, histology, genomic, and clinical data, demonstrating that cross-modal integration improves predictive performance and enables the discovery of novel biomarkers and therapeutic targets [23]. Boehm et al. further emphasized that multimodal data integration represents a necessary evolution beyond genomics-only approaches, although current limitations in dataset size and completeness remain a challenge for optimal model development [24].

Within hematologic malignancies, the integration of clinical variables, genomic features, and imaging data enables improved disease classification, clustering, and outcome prediction [17]. Machine learning algorithms are uniquely positioned to extract meaningful patterns from these complex, high-dimensional datasets, revealing interactions that are not accessible through traditional analytical methods [17]. As data availability and computational methods continue to advance, multi-modal integration is expected to play a central role in the development of next-generation precision hematology frameworks.

### 4.3. Advances in Computational Architectures

The computational methods underpinning dynamic and multi-modal artificial intelligence have evolved substantially in recent years. Dimensionality reduction and clustering techniques, such as t-distributed stochastic neighbor embedding (t-SNE) and Uniform Manifold Approximation and Projection (UMAP), allow visualization and identification of complex patterns in high-dimensional biological data [38]. These approaches are particularly valuable in hematologic malignancies, where single-cell sequencing and high-dimensional cytometry datasets are increasingly used to characterize cellular heterogeneity and disease evolution.

Ensemble learning and deep learning approaches have demonstrated improved performance in classification and prognostic modeling across a range of hematologic applications [17,38]. Commonly used algorithms, including random forests, support vector machines, gradient boosting methods, and neural networks, enable the analysis of complex, non-linear relationships within clinical and molecular datasets [17]. Increasingly, hybrid modeling strategies that combine traditional statistical approaches with machine learning techniques are being adopted, leveraging the interpretability of conventional models alongside the predictive power of modern AI methods [17].

More recently, advanced architectures such as transformer-based models and foundation models have expanded the scope of computational capabilities in biomedical AI. These models are pretrained on large-scale datasets and can be adapted to a wide range of downstream clinical tasks, including outcome prediction, biomarker discovery, and treatment response modeling [30]. In oncology, transformer-based and multimodal learning frameworks have demonstrated the ability to integrate diverse data modalities and capture complex relationships across biological scales [23,30].

In addition, graph-based learning approaches are emerging as powerful tools for modeling relational biological data, including gene–gene interactions, cellular networks, and tumor microenvironment dynamics [39]. These methods are particularly relevant for hematologic malignancies, where disease biology is inherently structured and interconnected across multiple levels of organization. Collectively, these advances in computational methods provide the technical foundation for the development of dynamic, multi-modal AI systems capable of addressing the complexity of hematologic diseases.

*Foundation Models and Large Language Models.* A more specific and rapidly evolving subset of transformer-based architectures, foundation models, warrants dedicated discussion given their growing prominence and direct relevance to hematologic oncology and cellular therapy. Foundation models are large-scale, self-supervised systems pretrained on extensive, often multimodal, datasets that can subsequently be adapted to numerous downstream clinical tasks, including outcome prediction, biomarker discovery, and treatment response modeling, with limited task-specific labeled data [30]. This paradigm is particularly attractive in hematologic malignancies, where individual centers, and even multicenter consortia, frequently lack sufficient annotated data for rare disease subtypes. In oncology more broadly, transformer-based and multimodal foundation model frameworks have demonstrated the ability to integrate diverse data modalities, including radiology, histology, genomic, and clinical data, and to capture complex relationships across biological scales, improving predictive performance and enabling discovery of novel biomarkers [23,30]. Within hematology specifically, foundation model architectures remain in early stages of adaptation, but their capacity to learn generalizable representations from large pretraining corpora is conceptually well suited to hematolymphoid diagnostics, where diagnostic variability outside specialized referral centers has already been identified as a key limitation of current, narrowly trained AI approaches [3].

Large language models (LLMs) represent a further extension of the foundation model paradigm into unstructured clinical text and, increasingly, multimodal clinical decision support. Within hematology, LLM-based clinical decision support systems (CDSS) have emerged as one of the most prominent trending applications in the recent literature, reflecting growing recognition of their potential value in direct patient care as massive amounts of pre-recorded longitudinal electronic health record data become available for these models to draw upon [19]. However, systematic reviews continue to note inconsistencies in LLM performance within oncology-facing CDSS, and prospective validation of LLM-based and broader AI-driven decision support within dedicated hematology and cellular therapy workflows remains largely absent from the literature [31]. More recent efforts have sought to ground LLM outputs in structured clinical knowledge to mitigate these limitations; CliCARE, for example, grounds large language model outputs in clinical practice guidelines to support decision-making over longitudinal cancer electronic health records [31]. This architectural strategy of grounding LLMs in curated clinical guidelines and structured data, rather than relying on general-purpose pretraining alone, may be necessary to achieve clinically acceptable performance in the high-stakes, guideline-driven setting of hematologic malignancy management.

For cellular therapy specifically, multimodal foundation models capable of jointly reasoning over clinical notes, laboratory trends, imaging, flow cytometry, and manufacturing data represent a natural extension of the adaptive, longitudinal paradigm proposed throughout this review; such systems could, in principle, continuously synthesize the heterogeneous data streams described in Section 6 (manufacturing quality metrics, immune reconstitution kinetics, toxicity grading) into unified, updatable risk assessments. However, no foundation model or LLM-based system has yet been developed or validated specifically for conditioning, lymphodepletion, or GVHD/CRS/ICANS management, and important challenges, including limited hematology-specific training and validation data, black-box reasoning, and the absence of regulatory frameworks for generative and adaptive AI systems, must be addressed before these tools can be responsibly deployed at the bedside [19,30,31,40,41].

### 4.4. Explainable and Interpretable AI

The importance of transparency in clinical decision support systems cannot be overstated, particularly in high-stakes settings such as hematologic malignancy management [42]. Explainability has emerged as a critical requirement for the safe and effective deployment of artificial intelligence in healthcare, with implications spanning technological, clinical, ethical, and legal domains. Amann et al. provided a comprehensive multidisciplinary assessment of explainability in medical AI, emphasizing that the absence of interpretability may undermine core ethical principles, including autonomy, beneficence, and accountability [42].

Explainability methods help identify key predictive features, improve clinician trust, and support biological interpretation of model outputs [42]. From a technological perspective, these methods provide insight into how models generate predictions, enabling validation and refinement. From a clinical perspective, they facilitate alignment between model outputs and established medical knowledge, which is essential for adoption in practice. Legal and regulatory considerations, including informed consent, certification of AI systems as medical devices, and assignment of liability, further underscore the necessity of interpretable models [42].

Specific explainability techniques, such as Shapley Additive Explanations (SHAP) and Local Interpretable Model-Agnostic Explanations (LIME), are increasingly applied to interpret predictions in oncology and hematology, enabling identification of the clinical, molecular, or imaging features driving individual predictions [43]. These approaches allow clinicians to assess whether model outputs are biologically plausible and clinically meaningful, thereby supporting trust and adoption.

However, the “black-box” nature of many advanced machine learning models remains a barrier to clinical implementation. The development of standardized frameworks for evaluating and reporting explainability, as well as integration of interpretability into model design, represents an important priority for future research. Ensuring that AI systems are both accurate and interpretable will be essential for translating computational advances into real-world clinical impact.

### 4.5. Data Augmentation and Synthetic Data

Synthetic datasets generated using generative artificial intelligence models have emerged as a promising approach to address critical challenges in hematologic malignancy research, including limited sample sizes, class imbalance, and privacy constraints [16]. These limitations are particularly relevant in hematology, where rare disease subtypes and heterogeneous patient populations often restrict the availability of large, well-annotated datasets for model development.

D’Amico et al. demonstrated that conditional generative models can be used to generate realistic synthetic clinical-genomic datasets in myelodysplastic syndromes and acute myeloid leukemia, providing a framework for data augmentation while preserving key statistical and biological properties of the original data [16]. Importantly, such approaches enable the expansion of limited datasets and can support the development of predictive models in settings where real-world data are scarce or fragmented.

The potential applications of synthetic data in hematology are broad. These include model training, hypothesis testing, simulation of clinical scenarios, and rare disease modeling [16]. In addition, synthetic datasets may facilitate the development and validation of machine learning models while reducing concerns related to patient privacy and data sharing.

Beyond hematology, generative approaches such as generative adversarial networks (GANs) have been applied to create large-scale synthetic patient cohorts for training decision-support systems, demonstrating that synthetic data augmentation can enable robust model development even in data-constrained environments [44]. These findings highlight the potential of synthetic data to accelerate research and support the implementation of AI-driven precision medicine.

Despite these advances, challenges remain, including ensuring fidelity to real-world data, avoiding the propagation of biases, and establishing standardized validation frameworks. Addressing these limitations will be essential to ensure that synthetic data approaches can be safely and effectively integrated into clinical and research applications.

### 4.6. Federated and Distributed Learning

Federated learning (FL) enables multi-institutional model development without sharing raw patient data, addressing critical regulatory and privacy challenges in healthcare AI implementation [17]. In this framework, model parameters, rather than the underlying data, are shared during training, allowing institutions to collaboratively develop predictive models while maintaining data sovereignty and patient confidentiality. Shouval et al. highlighted federated learning as an important emerging approach in hematology, noting its potential to improve generalizability across populations and practice settings by leveraging distributed datasets [17].

Federated learning has demonstrated substantial promise across oncology applications. Multi-institutional studies have shown that federated approaches can achieve performance comparable to centrally trained models while preserving data privacy, supporting their use in collaborative clinical research environments [45]. In addition, systematic evaluations of federated learning in cancer research have demonstrated improved robustness and generalizability across diverse datasets and clinical contexts [46].

This approach is particularly relevant for hematologic malignancies, where many disease subtypes are rare and individual institutions may lack sufficient data to develop robust predictive models. Federated learning enables the aggregation of knowledge across centers without requiring centralized data sharing, thereby overcoming key barriers related to data fragmentation and regulatory constraints [17].

Despite its advantages, federated learning introduces new challenges, including communication efficiency, model heterogeneity across institutions, and the need for advanced privacy-preserving techniques such as differential privacy and secure aggregation. Addressing these challenges will be essential for translating federated learning from research settings into routine clinical practice.

### 4.7. Reinforcement Learning for Adaptive Treatment Strategies

Reinforcement learning (RL) addresses sequential decision-making tasks by exploring the underlying dynamics of an environment and taking actions to maximize cumulative rewards over time, making it conceptually well suited for optimizing dynamic treatment regimens in oncology [47]. Unlike supervised learning, which primarily predicts outcomes from predefined inputs, RL learns optimal policies through sequential interactions, enabling adaptive and longitudinal treatment optimization.

Q-learning, a leading RL approach, has been applied to develop personalized adaptive treatment strategies from registry and cohort data. Krakow et al. demonstrated the use of Q-learning on Center for International Blood and Marrow Transplant Research registry data to identify optimal sequences of immunosuppressant classes for graft-versus-host disease prophylaxis and treatment, illustrating how RL can guide individualized sequential treatment decisions in the transplant setting [25]. This approach is directly relevant to cellular therapy, where sequential decisions regarding conditioning, immunosuppression, and toxicity management must be made over time.

Deep reinforcement learning extends these capabilities by using neural networks to approximate value functions in high-dimensional state and action spaces. DRL-based frameworks have been developed for adaptive chemotherapy dosing, where agents learn dosing strategies intended to inhibit tumor growth while minimizing damage to normal tissues [48]. Engelhardt and Michor further highlighted the complementary roles of mathematical modeling and machine learning in constructing dynamic optimal cancer treatment strategies, emphasizing the potential of adaptive computational approaches in precision oncology [49].

The application of RL to cellular therapy settings, including optimization of conditioning intensity, lymphodepletion regimens, immunosuppression protocols, and toxicity management, remains largely unexplored but represents a high-priority area for future development. The sequential, multi-step nature of cellular therapy management aligns naturally with the RL framework, particularly as patient status evolves from pre-treatment assessment through infusion, early toxicity monitoring, and long-term response evaluation.

### 4.8. Digital Twins in Hematologic Oncology

Digital twin technology, originally developed in engineering disciplines, has emerged as a promising concept in oncology. A digital twin represents a dynamic, virtual replica of a patient’s physiological and pathological state, integrating multi-dimensional data to enable personalized simulation of disease progression and treatment response [27]. By combining clinical, molecular, and imaging data within computational models, digital twins offer the potential to support individualized treatment planning and optimization.

In oncology, digital twin frameworks are being explored across multiple domains, including precision treatment selection, therapy optimization, drug development, and modeling of treatment response [27,28]. These approaches aim to simulate patient-specific disease trajectories and evaluate potential therapeutic strategies in silico before clinical implementation. In hematology, such capabilities are particularly attractive given the complex, multi-omic nature of diseases such as MDS and AML.

The integration of artificial intelligence with multi-omics data, imaging biomarkers, and longitudinal clinical information is central to the development of digital twin models [27,29]. These systems have the potential to continuously update as new patient data become available, enabling real-time refinement of predictions and treatment strategies. This aligns closely with the broader shift toward dynamic, data-driven clinical decision-making frameworks.

However, digital twin technology in oncology remains largely in early stages of development. Current applications are predominantly preclinical or proof-of-concept, with limited real-world clinical implementation [28,29]. Key challenges include data integration across heterogeneous sources, model validation, computational scalability, and ethical considerations related to data use and decision-making. Despite these limitations, digital twins represent a compelling future direction for precision hematology. Their ability to integrate multi-modal data, simulate disease evolution, and support personalized treatment optimization positions them as a natural extension of dynamic, multi-modal AI systems.

### 4.9. Toward Real-Time Clinical AI Systems

The convergence of dynamic modeling, multi-modal data integration, and advanced computational architectures is driving the development of real-time clinical artificial intelligence systems. These systems are designed to integrate with electronic health records and digital infrastructure, enabling continuous updating of predictions based on incoming patient data and shifting the paradigm from retrospective modeling toward prospective clinical decision support [35].

The increasing availability of digitized health data, including electronic health records, genomic datasets, and imaging, provides the foundation for AI systems capable of operating in real time, generating actionable insights at the point of care [35,50]. Rather than relying on static risk estimates at diagnosis, real-time systems can continuously reassess patient status as new clinical information becomes available, allowing for dynamic risk stratification and adaptive management strategies [2,17].

Early examples of this paradigm are reflected in dynamic modeling approaches applied to hematologic malignancies. Models developed by Bobak et al. and Nazha et al. demonstrate that continuous risk reassessment using longitudinal clinical and molecular data is both feasible and clinically informative [22,32]. These approaches illustrate how real-time updating of predictions can enhance clinical decision-making across the disease course.

Integration with electronic health record systems is a critical prerequisite for the deployment of real-time AI in clinical practice. Embedding AI-driven decision support within clinical workflows enables timely, context-aware recommendations that can support clinician decision-making [35]. However, significant challenges remain, including the need for prospective validation, standardized data infrastructure, regulatory frameworks, and demonstration of clinical utility and impact on patient outcomes [2,17]. As these barriers are addressed, real-time clinical AI systems are expected to play an increasingly central role in precision medicine, enabling continuous, data-driven optimization of patient care in hematologic malignancies.

### 4.10. Relevance to Hematological Malignancies and Cellular Therapies

The emerging paradigm of dynamic, multi-modal artificial intelligence is particularly well suited to hematologic malignancies, which are characterized by complex biology, multi-omic drivers, and highly dynamic treatment trajectories [2,17,36]. Diseases such as myelodysplastic syndromes, acute myeloid leukemia, lymphoma, and multiple myeloma exhibit substantial heterogeneity at the molecular, cellular, and clinical levels, necessitating analytical approaches capable of integrating diverse data types and capturing temporal evolution. This paradigm is especially relevant for optimizing advanced therapies, including chimeric antigen receptor T-cell (CAR-T) therapy and allogeneic hematopoietic stem cell transplantation. These interventions require dynamic decision-making across multiple time points, including conditioning regimen selection, lymphodepletion strategies, infusion parameters, immunosuppression management, and toxicity monitoring. Current approaches remain largely protocol-driven, with limited personalization of these critical treatment variables [2,17].

Dynamic AI systems that integrate longitudinal clinical data with multi-modal molecular and imaging features offer the potential to enable individualized optimization of these complex treatment pathways. For example, real-time integration of evolving biomarkers, such as cytokine profiles, immune reconstitution dynamics, and measurable residual disease, could support adaptive decision-making in the prevention and management of complications such as cytokine release syndrome or graft-versus-host disease. Reinforcement learning frameworks provide a conceptual and methodological foundation for such adaptive treatment strategies. The application of Q-learning to transplant-related decision-making, as demonstrated by Krakow et al., illustrates how sequential treatment optimization can be achieved using real-world clinical data [25]. Extending these approaches to cellular therapies represents a promising direction for future research.

### 4.11. Key Implications

Dynamic, multi-modal, and interpretable artificial intelligence systems represent a necessary evolution toward clinically actionable tools capable of guiding real-time management decisions in hematologic malignancies [2,16,17,35,36,38,42]. The transition from static, baseline-only models to temporally dynamic systems that incorporate longitudinal data streams, multi-modal data integration, and advanced computational architectures reflects a fundamental shift in how clinical prediction and decision support are conceptualized. The integration of explainable AI, synthetic data generation, and distributed learning frameworks further enhances the feasibility of deploying these systems in real-world clinical settings. Together, these advances enable not only improved predictive performance but also greater transparency, scalability, and generalizability across diverse patient populations [16,17,42].

**Figure 3:**
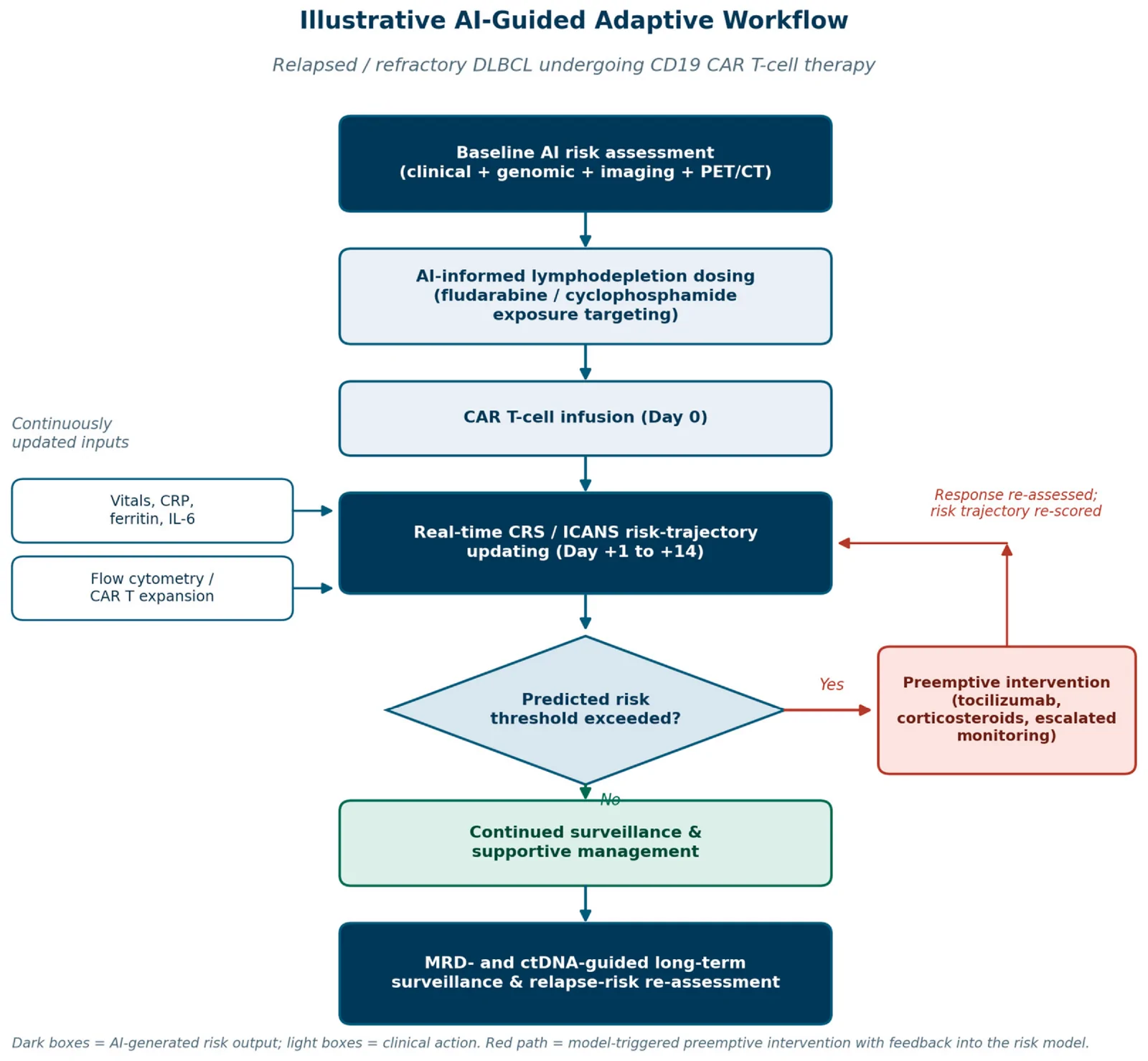
Illustrative AI-guided adaptive workflow for a hypothetical patient with relapsed/refractory diffuse large B-cell lymphoma (DLBCL) undergoing CD19-directed CAR T-cell therapy, tracing baseline multimodal risk assessment through AI-informed lymphodepletion exposure targeting, infusion, and real-time CRS/ICANS risk-trajectory updating driven by continuously acquired vital sign, inflammatory marker, and CAR T-expansion data. Crossing a predicted risk threshold triggers preemptive intervention, the response to which is fed back into the model for risk re-scoring (red path), distinguishing this closed-loop architecture from the static, single-timepoint prediction that characterizes most currently published models. Dark boxes denote AI-generated risk output and light boxes denote clinical action. This workflow is a conceptual illustration; no component has yet been prospectively validated in a clinical trial.

Importantly, emerging dynamic modeling approaches demonstrate that continuous risk reassessment using longitudinal clinical and molecular data is both feasible and clinically informative [22,32]. These developments support a transition from static prediction toward adaptive, data-driven clinical decision-making frameworks that evolve alongside the patient’s disease course. As digital infrastructure, data availability, and computational methods continue to advance, AI-driven risk stratification and treatment optimization are poised to play an increasingly central role in hematologic malignancies. The successful translation of these technologies into clinical practice will depend on interdisciplinary collaboration, rigorous validation, and a sustained focus on developing systems that are not only accurate, but also interpretable, trustworthy, and clinically actionable. An illustrative example of this adaptive, closed-loop approach in the CAR T-cell setting is shown in Figure 3.

## 5. AI-Guided Adaptive Therapy in Cellular Therapies

The integration of artificial intelligence (AI) into the management of hematologic malignancies is shifting from static prognostic scoring toward dynamic, data-driven clinical decision support. Conventional risk models rely on variables captured at a single time point, but machine learning (ML) now permits dynamic risk assessment through the modeling of time-series data, a substantive advance over traditional snapshot-based approaches [26] (Representative studies illustrating this shift are summarized in Table 3).

**Table 3:** Representative AI/ML Studies in Hematologic Malignancies and Cellular Therapy.

Study/Model	Application	Data Modality	Key Finding	Refs.
Alem et al.	Outcome prediction across hematologic cancers	Clinical, laboratory	Pooled AUC 0.78–0.80 overall; externally validated top-performing algorithms reached AUC 0.87 for treatment-related complications, outperforming standard regression models.	[1]
MOSAIC (D’Amico et al.)	Multimodal classification and prognostic assessment in MDS	Clinical + genomic (multimodal)	Nonlinear AI survival prediction outperformed conventional statistical/reference tools; maintained performance across institutions via federated learning; incorporated explainable AI.	[13]
Bobak et al.	Dynamic mortality risk prediction in MDS	Longitudinal clinical data	AUC ~0.80 in independent validation; outperformed static baseline models.	[32]
Nazha et al.	Personalized survival/risk stratification in MDS	Clinical + molecular	Random survival forest model dynamically updates survival probabilities at successive clinical time points.	[22]
Shimomura et al.	Conditioning intensity selection (MAC vs. RIC) in MDS transplantation	Clinical/transplant variables	ML-derived benefit score identified patients with superior PFS from RIC vs. MAC and vice versa, enabling personalized intensity selection beyond categorical assignment.	[33]
PKU-AML model (Fan et al.)	Post-transplant relapse prediction after HID-HSCT in AML	Disease risk, treatment history, MRD status, donor characteristics	Externally validated; predictive accuracy exceeded unaided clinical judgment.	[9]
D’Amico et al.	Synthetic data augmentation for MDS/AML model training	Clinical + molecular (synthetic, GAN-generated)	300% augmented synthetic cohort (from *n* = 7133) anticipated molecular classification/scoring systems later derived from much larger real-world cohorts.	[2,16]
Mushtaq et al.	GVHD prediction and adaptive management	Clinical, immunologic	Q-learning enabled adaptive immunosuppression strategies for acute/chronic GVHD beyond static tapering; ML outperformed multivariable statistical models in donor selection.	[26]

This evolution is most clearly illustrated by the development of adaptive treatment strategies using reinforcement learning methods such as Q-learning, in which an algorithm iteratively learns optimal treatment decisions by modeling sequential clinical states and outcomes as new patient data become available [26]. Practical frameworks for the validation, risk classification, and regulatory oversight of AI-based tools in clinical laboratory settings are also being established, providing an initial scaffold for safe deployment [15].

Both CAR-T therapy and allogeneic HSCT possess features that make them well suited to adaptive AI approaches. They have a narrow therapeutic window, substantial toxicity risk, and a dynamic post-treatment course. In the CAR-T setting, lymphodepletion is essential for engraftment and persistence, yet dosing schemes vary widely across institutions and clinical trials [11]. In the allogeneic HSCT setting, GVHD remains among the most consequential complications, contributing to significant morbidity and mortality [26]. Management of both therapies remains largely standardized rather than individualized, and although ML models have shown promise in predicting hematological cancer outcomes, existing studies generally exhibit a high risk of bias, highlighting the distance between predictive capability and clinical implementation [1].

Currently, conditioning regimen selection in allo-HSCT relies on clinical factors such as patient age, comorbidities, and disease type, with intensity categorized broadly as myeloablative (MAC) or reduced-intensity (RIC). Shimomura et al. demonstrated that ML can move beyond this categorical approach by developing a benefit score for RIC versus MAC in patients with MDS; in validation, the model identified patients who derived significantly better progression-free survival from RIC versus MAC and vice versa, illustrating that ML-based scoring can personalize conditioning intensity selection. More broadly, ML approaches have demonstrated potential in improving conditioning regimen decisions alongside donor selection and post-transplant outcome prediction, though challenges in data standardization, interpretability, and ethical considerations persist [33].

Lymphodepletion before CAR-T cell infusion currently relies on fixed fludarabine (75–120 mg/m^2^) and cyclophosphamide (750–1500 mg/m^2^) regimens, but substantial inter-patient variability in pharmacodynamics and clinical response has been well documented [3]. While no AI-based tool has yet been developed specifically to optimize lymphodepletion dosing and timing, the demonstrated capacity of AI models to integrate multivariate clinical data in the transplant setting suggests that AI-guided personalization of lymphodepletion is a logical area for future development [9,13,33].

Among the most compelling applications of adaptive AI in allo-HSCT is the dynamic modulation of immunosuppression. Q-learning has been applied to develop adaptive treatment strategies for the personalized prevention and treatment of both acute and chronic GVHD, moving beyond static immunosuppression tapering by modeling time-series data and enabling therapeutic intensity to be adjusted in response to evolving clinical signals [26]. ML algorithms have also demonstrated superiority over traditional multivariate statistical models in donor selection for allo-HSCT, and deep learning models can accurately identify tissue affected by chronic GVHD [26]. Realizing the full potential of these approaches will require large-scale, multicenter collaborations to develop generalizable models [26].

Current management of treatment-related complications, including CRS, ICANS, and GVHD, remains largely reactive. Emerging predictive modeling may enable a shift toward preemptive strategies; in a meta-analysis by Alem et al., externally validated ML algorithms achieved a pooled AUC of 0.87, significantly outperforming standard regression [1]. Together with the disease-specific models described above, these findings suggest that ML-based risk stratification could facilitate identification of high-risk patients before clinical deterioration and support preemptive interventions such as early cytokine blockade, corticosteroid initiation, or intensified monitoring.

AI-based models are beginning to integrate multiple clinical and disease-related variables for post-transplant outcome prediction. The PKU-AML model by Fan et al., developed and externally validated in patients with AML receiving HID HSCT, combined disease risk, treatment history, MRD status, and donor characteristics to predict post-transplant relapse with accuracy exceeding unaided clinical judgment [9]. The MOSAIC framework extended this approach by applying deep learning to integrate clinical and genomic features for survival prediction in MDS, incorporating explainable AI methods to enhance interpretability and demonstrating through federated learning that high model performance can be maintained across institutions without centralizing sensitive patient data [13]. To address the data scarcity that limits training of such models, D’Amico et al. separately showed that synthetic data generation using conditional generative adversarial networks in a cohort of 7133 patients with MDS/AML can augment existing datasets; a 300% augmented synthetic cohort anticipated molecular classification and scoring systems obtained years later from substantially larger real-world cohorts. While these models demonstrate the feasibility of multimodal risk stratification, none have yet been translated into clinical practice [2,16].

Translating these tools into clinical practice faces several barriers. Existing ML studies generally exhibit high risk of bias, and model superiority over standard regression has emerged only upon external validation rather than in internal testing, underscoring the importance of adherence to standardized risk-of-bias assessment frameworks in future model development [1]. No ML-based pipeline in myeloid neoplasms has been directly translated into clinical practice, reflecting persistent gaps in regulatory frameworks, workflow integration, and model interpretability [11]. Prospective validation in clinical trials is notably absent and will be essential to establish whether these models can reliably guide treatment decisions in real-time clinical settings [11]. Clinician unfamiliarity with ML tools and patient concerns regarding privacy and reliability of outcomes remain additional obstacles, and the implementation of stringent ethical guidelines is a prerequisite for broader adoption [2,26].

AI-guided adaptive therapy has the potential to enable personalized, real-time optimization of treatment strategies across the continuum from conditioning and lymphodepletion through post-treatment immunosuppression and toxicity management in cellular therapies. The models and frameworks described here, which span relapse prediction, multimodal classification with federated implementation, reinforcement learning-based adaptive treatment strategies, predictive models with superior external discriminative ability, and synthetic data technologies collectively provide a substantive technical foundation [1,2,9,13,15,16,26]. Translation into clinical practice will require prospective validation, regulatory clarity, clinician engagement, and resolution of interpretability and privacy concerns [2,11].

## 6. Implementation and Clinical Integration of Artificial Intelligence

The translation of AI from predictive modeling to real-time clinical integration represents a current and consequential challenge in the field. Despite a rapidly growing breadth of literature demonstrating the discriminative performance of ML models in diagnostics and management of hematological malignancies, few AI tools have been successfully deployed into clinical workflows. A 2025 comprehensive review in *Blood* reported that AI tools remain infrequently used in clinical practice, with key barriers including data quality concerns and/or unclear avenues for quality assurance, limited infrastructure support for AI deployment, and lacking regulatory frameworks [20]. In the context of stem cell transplant and cellular therapies, these barriers are amplified. The complexity of relevant data streams should be expanded beyond staging and treatment to include genomics and/or cellular compatibility, manufacturing quality assurance, pharmacokinetics, immune reconstitution, and longitudinal toxicity monitoring [51]. The computational approaches needed to synthesize these expanded data streams are not yet at scale for a typical clinical practice.

A foundational prerequisite for AI utilization in clinical practice is seamless integration into the electronic health record (EHR). Many AI models within and beyond hematology are not directly integrated into the EHR and require time-consuming manual data entry or query design which easily disrupts clinical workflows [20]. Additionally, data heterogeneity between institutions and/or EHR data centers creates inconsistencies that limit model portability and generalizability at baseline [18,51]. This conflict is particularly noticeable in the case of cellular therapies, in which multicenter collaboration is indispensable. The Fast Healthcare Interoperability Resources (FHIR) protocol has emerged as a standardization framework to enable structured electronic exchange of clinical data, and large-scale oncology consortium initiatives such as EuCanImage have demonstrated that implementing consistent data capture workflows using validated tools can yield interoperable, AI-ready repositories at scale [52]. However, in the cellular therapy setting specifically, standardized, comprehensive data repositories beyond those which require manual data contributions, such as the Center for International Blood and Marrow Transplant Research (CIBMTR) data repository [53], are required to enable meaningful AI learning across myriad data collected in the provision of these specialized therapies [51].

In addition to EHR adoption, embedding AI tools within point-of-care clinical decision support systems (CDSS) is increasingly recognized as a prerequisite for clinical uptake, as standalone models that exist outside of clinical workflows are unlikely to influence real-time decisions. In hematology, CDSS have emerged as one of the most prominent trending applications in the recent literature [19]. The specific promise and current limitations of large language model-based CDSS are discussed in Section 4.3; from an implementation standpoint, the operative constraint is that prospective validation of AI-driven CDSS within dedicated hematology and cellular therapy workflows remains largely absent from the literature [31].

Given the rarity of hematological malignancies within the greater practice of medicine, the datasets any one health system can generate are limited. To combat this, federated learning (FL) has emerged as a strategic implementation architecture between institutions. FL enables collaborative model training across multiple institutions by exchanging model parameters rather than raw patient data, thereby preserving data privacy while harnessing the statistical power of distributed, multisite datasets [54]. As demonstrated by the MOSAIC framework applied to myelodysplastic syndromes, high model performance can be maintained across geographically dispersed institutions without centralizing sensitive patient data, establishing federated approaches as both technically feasible and ethically aligned for hematologic malignancy research [13]. However, real-world implementation of FL in hospital environments encounters persistent challenges which remain incompletely addressed, including inter-institutional collaboration barriers, the need for standardized FL protocols, and the ethical and logistical complexities of obtaining patient consent for federated model training [55].

An underappreciated but high-impact domain for AI clinical integration in cellular therapy is the manufacturing pipeline itself. Hematopoietic stem cell and CAR-T cell production are complex, multi-step biological processes characterized by significant batch-to-batch variability. Over the past decade, the high-throughput data generated during CAR-T manufacturing, encompassing single-cell RNA sequencing, cytometry by time-of-flight (CyTOF), spatial transcriptomics, metabolomics, and proteomics, has expanded dramatically, and the meaningful interpretation of these datasets increasingly requires AI-driven analysis [51]. Integrating AI models trained on manufacturing data with downstream clinical outcome data creates a closed-loop opportunity to identify the product characteristics most predictive of durable clinical response, enabling iterative quality improvement at the manufacturing level and prospective patient selection for cellular therapy [51]. Furthermore, the application of AI to CAR design itself, including target antigen identification, vector architecture optimization, and prediction of T-cell differentiation trajectories, represents additional dimensions of manufacturing integration where ML approaches are actively being explored [56]. Despite the conceptual appeal and growing technical feasibility of these manufacturing-to-clinical integration strategies, no AI pipeline that spans the full continuum from manufacturing quality metrics to post-treatment outcome has yet been prospectively validated in a clinical CAR-T program, and the regulatory pathway for such integrated AI-manufacturing tools remains to be clearly defined [51].

## 7. Challenges and Limitations

Despite these substantial advancements in AI model development, the transition from research prototype to clinical tool in hematological malignancies and cellular therapy remains stalled by technical, structural, and ethical barriers. From a technical standpoint, the relative rarity of hematological malignancies presents a persistent scarcity of high-quality, easily accessible data sets to train these models on, even when assisted with FL systems [20,51]. Molecular data in particular are not equally available across patient populations, as EHR systems frequently fail to incorporate genomic variables in a structured, queryable format, and the absence of standardized ontologies across institutions further compounds data sparsity [1]. Generative AI approaches such as conditional generative adversarial networks have shown promise in augmenting limited hematologic datasets while preserving statistical properties of the source data, and synthetic cohorts have demonstrated concordance with molecular classification systems derived years later from substantially larger real-world datasets [40]. Nevertheless, the regulatory and methodological frameworks governing the use of synthetic data for model training in clinical AI development remain nascent.

As previously mentioned, the “black box” problem, or the development of deep learning architectures that are unable to share the reasoning behind their predictions, persists as another important technical limitation [40]. For both clinicians and patients to build trust in AI-based clinical decision support systems, model outputs must not only be accurate but also explainable in terms that map onto recognizable clinical reasoning [40]. Explainable AI (XAI) has emerged as a dedicated field to address this gap, with several reviews suggesting that explainability may not only build clinical trust but also generate new biological insight [16,40]. However, accuracy-interpretability trade-offs persist; simpler, more interpretable models frequently underperform complex deep learning architectures, and no consensus standard for evaluating the clinical adequacy of AI explanations currently exists [57].

Beyond these technical limitations lies an AI regulatory landscape that has not kept pace with the rate of model development. Traditional medical device approval frameworks are typically applied to AI tools with poor results, as their static nature does not fit the dynamic, evolving capabilities of most AI systems [41]. The absence of a clear regulatory pathway creates uncertainty for developers and clinicians alike, disincentivizing investment in prospective clinical trials that would generate the evidence required for regulatory clearance. Prospective validation in clinical trials is notably absent across the hematologic malignancy AI literature, and without it, the real-world clinical performance of models with promising retrospective metrics cannot be assumed [1]. Recommendations for standardized implementation of AI in clinical settings such as those employed in flow cytometry represent initial regulatory concepts, but guidance for HSCT and CAR-T AI tools is largely absent [15].

The ethical dimensions of AI deployment in hematology and cellular therapy are increasingly debated, as valid arguments can be made for and against an expanded role of AI in these high-stakes clinical contexts. Those in favor of rapid AI expansion argue that it has the potential to democratize access to specialized experts otherwise gatekept from smaller institutions. Furthermore, the capacity of AI to process and integrate data streams at a scale and speed that vastly exceeds human cognition represents a qualitatively distinct capability that, when properly validated and integrated, could materially improve outcomes for patients with relapsed or refractory hematological malignancies who otherwise face extremely limited options [7,51]. The potential for AI to accelerate hematology, cellular therapy, and medicine overall remains seemingly endless and may thereby impose an ethical imperative to utilize these tools to enhance biomedical progress and deliver results faster to patients.

Despite these benefits, substantial concerns have been raised regarding algorithmic bias and its potential to perpetuate or amplify existing healthcare inequities. Training datasets in hematology and oncology are drawn disproportionately from academic medical centers in high-income countries, and racial and ethnic minority populations are systematically underrepresented [40,58]. Studies have found that minority groups were absent from more than 90% of some medical datasets, and race and ethnicity data are frequently excluded or inconsistently recorded in EHRs, limiting the ability of AI models to learn patterns relevant to those populations or to be appropriately validated in them [58]. In the cellular therapy setting specifically, social determinants of health are an unavoidable reality of these costly and intense treatment regimens. Without proper programming on how to recognize these factors and associated biases against patients, AI systems are at risk of inheriting the mistakes of their human predecessors and broadcasting them through unprecedented means.

Questions of accountability and legal liability represent another contested domain in the ethics of clinical AI. When an AI-assisted decision leads to patient harm, through misdiagnosis, incorrect risk calculation, a miscalibrated toxicity model, or a flawed treatment recommendation, the allocation of responsibility between developers, deploying institutions, and clinicians remains unsolved [21,41]. This ambiguity creates a structural disincentive to clinical deployment: clinicians may be reluctant to act on AI recommendations for fear of liability if outcomes are poor, while developers may be reluctant to seek regulatory clearance given the absence of clear liability frameworks [21]. Patient autonomy and informed consent introduce additional complexity, as patients have a right to not only understand what treatments are recommended but the basis on which those recommendations are generated [59]. The opacity of the black-box model may further complicate this informed consent process, as even the users in some cases are unable to explain how their AI model arrived at its clinical reasoning. From the patient perspective, concerns about data privacy, bias, consent, and the potential loss of personal autonomy in medical decision-making are well-documented, while from the clinician’s perspective, AI may support decision-making but cannot replicate the nuance, empathy, and individualized contextual judgment that characterizes high-quality clinical care [60]. A balanced approach requires AI to function as a tool that augments rather than supplants clinician judgment, with the clinician retaining ultimate decision authority and ethical accountability [59,60].

Privacy represents an additional critical concern in the AI ecosystem for hematology and cellular therapy. The data streams required to train AI models are often subject to compliance with the Health Insurance Portability and Accountability Act (HIPAA), and are potentially high-value targets for privacy breaches [51]. Federated learning architectures, as described above, represent a partial technical solution; however, research has demonstrated that shared model parameters themselves may be vulnerable to inference attacks by untrusted aggregators, and that even federated systems do not provide absolute privacy guarantees without additional safeguards such as differential privacy or homomorphic encryption [61]. Furthermore, the question of who owns AI-generated insights derived from patient data, and may benefit financially from their commercialization, remains ethically contested.

Finally, the impact on the hematology and cellular therapy workforce is understated but essential to conceptualizing the ethical arguments surrounding AI. A substantial proportion of hematologists and oncologists report limited familiarity with AI tools, and even well-explained model outputs may lead to misinterpretation without appropriate training in AI literacy [20,40]. Medical education has not yet systematically incorporated AI literacy into curricula, and clinicians who lack foundational understanding of the strengths and limitations of ML models are poorly positioned to use AI decision support critically or to recognize when model outputs should be questioned [40]. Additional concerns about “skill atrophy” from overreliance on AI models by students, residents, and fellows remain an area of concern as there are dueling imperatives to both introduce and shield trainees from AI systems. Ultimately, the implementation of stringent ethical governance frameworks, including periodic re-certification of deployed AI systems, multistakeholder oversight committees, and ongoing monitoring for performance drift and inequitable outcomes, is a prerequisite for responsible and sustainable AI integration in the clinical care of patients with hematological malignancies and beyond [41,60].

## 8. Future Directions

Future progress in artificial intelligence for hematologic malignancies will depend on prospective, ideally multicenter, studies evaluating whether AI-guided decision-support systems can improve clinical outcomes beyond retrospective performance metrics alone [62,63,64]. Accordingly, the field will likely require a transition from retrospective prediction toward interventional study designs in which AI outputs are explicitly linked to treatment modification, workflow integration, and patient outcomes [62,64,65].

A parallel priority is the development of adaptive systems that can continually incorporate EHR-derived variables, laboratory information contained within those records, and patient-generated digital data over time rather than relying on a single pretreatment snapshot [63,65]. In selected high-risk settings, this evolution may eventually support reinforcement learning and closed-loop decision-support approaches in which recommendations are repeatedly updated as new clinical information becomes available [65,66]. Expansion of multimodal and multi-omics integration will also be essential, because combining genomic, transcriptomic, proteomic, and additional biological datasets can improve understanding of disease biology and treatment response [65,67]. Such integration may reveal molecular patterns and disease-relevant pathways not apparent from any single modality alone [67]. In parallel, the emergence of foundation models and large language models illustrates a complementary trajectory toward generalizable, broadly pretrained clinical AI; realizing their potential in cellular therapy will require dedicated hematology-specific validation and grounding strategies that mitigate the reliability concerns already observed in oncology-facing decision support before these tools can be safely integrated with the adaptive, multimodal frameworks discussed throughout this review [19,30,31].

At the data-infrastructure level, federated learning offers a path to collaborative, multi-institutional model development without exchanging raw patient data, which may improve robustness and generalizability while addressing major privacy and data-governance barriers [68]. These federated ecosystems may be particularly valuable in hematologic malignancies and cellular therapies, where clinically important subgroups, rare toxicities, and center-specific practices can limit model development at any single institution [68].

Synthetic data generation represents another important future direction, because it may expand privacy-conscious development and validation workflows in data-limited settings while still requiring careful attention to privacy, bias, and regulatory oversight [69,70]. To influence practice, these models will need to be embedded into clinician-facing decision-support systems that fit routine clinical workflows and support safe human–AI interaction at the point of care [64,65]. Standardized data capture and reporting frameworks, including TRIPOD + AI for prediction models and CONSORT-AI for interventional trials, will be essential for transparent, reproducible, and clinically interpretable translation [34,64,71].

As AI systems become more adaptive and more deeply integrated into care, governance and regulatory frameworks will also need to evolve around accountability, transparency, privacy, and continuous oversight, while clinician education and collaboration with data scientists remain prerequisites for responsible adoption [62,65,68,71]. For cellular therapies specifically, the long-term opportunity is to use these advances to support increasingly individualized choices in conditioning, lymphodepletion, immunosuppression, toxicity prevention, and treatment sequencing, thereby shifting the field from prediction alone toward intervention-guiding systems [63,65,66,67,68,69,70]. Ultimately, meaningful progress will depend on the integration of robust data infrastructure, prospective validation, transparent reporting, and clinically actionable deployment strategies capable of improving patient outcomes in real time [34,62,64,68].

## 9. Conclusions

Hematologic malignancies and cellular therapies constitute uniquely heterogeneous and temporally dynamic clinical settings in which conventional baseline prognostic systems incompletely capture evolving disease biology, treatment response, relapse risk, and treatment-related toxicity [1,2,17]. Across these diseases, artificial intelligence has demonstrated substantial promise in diagnostic support, prognostic stratification, and multimodal data integration [1,13,17,23]. Emerging frameworks such as multimodal oncology models and MOSAIC illustrate how integrated analysis of clinical, genomic, imaging, pathology, and outcome data can generate more personalized and biologically informed predictions than single-modality approaches [1,13,17,23].

Despite these advances, the field remains constrained by the predominance of retrospective and largely static models, recurrent concerns regarding bias and generalizability, incomplete reporting of calibration and clinical utility, and the persistent failure of most published pipelines to achieve routine bedside implementation [1,2,15,17]. This translational gap is particularly important in cellular therapy, where clinically meaningful decisions regarding conditioning intensity, lymphodepletion, immunosuppression, toxicity surveillance, and relapse monitoring unfold sequentially over time [9,11,25,26,33,51]. These decisions therefore require support systems that can adapt as patient status changes rather than rely on a single pretreatment snapshot [9,11,25,26,33,51]. Cellular therapy is inherently longitudinal, whereas most current AI systems remain fundamentally cross-sectional [1,2,17,51]. The central challenge is therefore no longer whether AI can predict outcomes, but whether it can support real-time therapeutic intervention [1,2,15,17,51].

Published studies already point toward what a clinically integrated future could look like: machine-learning models have been used to individualize transplant conditioning intensity, predict post-transplant relapse, and frame adaptive treatment strategies for graft-versus-host disease [9,25,26,33], while CAR-T-focused work emphasizes the need to optimize lymphodepletion, toxicity prediction, and longitudinal management across the full therapeutic cycle [11,51]. The next priority for the field, therefore, is not merely improved predictive discrimination, but the development of systems integrated within clinical workflows that continuously incorporate longitudinal clinical, laboratory, molecular, and treatment data into clinical recommendations for treatment selection, toxicity prevention, and ongoing patient management [13,23,25,51,54].

Achieving that shift will require interoperable and comprehensive data repositories, privacy-preserving multicenter learning strategies, rigorous external and prospective validation, transparent reporting, and implementation pathways that demonstrate real-world clinical utility rather than algorithmic accuracy alone [1,13,15,51,54]. Equally important, adoption will depend on earning clinician and patient trust through interpretability, governance, and careful integration into clinical workflows so that AI functions as an adjunctive clinical tool in hematology practice rather than a poorly interpretable system [2,15,54]. Ultimately, the transition from predictive to intervention-guiding AI represents a critical step toward precision medicine in hematologic malignancies and cellular therapies, with the potential to improve outcomes while reducing treatment-related morbidity if these technologies are developed, validated, and implemented responsibly [1,9,11,25,33,51].

## Data Availability

No new data were created or analyzed in this study. Data sharing is not applicable to this article.
